# Autonomy Over Independence: Self-Determination in Catalonia, Flanders and South Tyrol in the Aftermath of the Great War

**DOI:** 10.1177/02656914231198182

**Published:** 2023-10-05

**Authors:** Emmanuel Dalle Mulle, Mona Bieling

**Affiliations:** Universidad Complutense de Madrid, Spain and Albert Hirschman Centre on Democracy, 30525 Graduate Institute of International and Development Studies, Geneva, Switzerland; 30525 Graduate Institute of International and Development Studies, Geneva, Switzerland

**Keywords:** Autonomy, nationalism, self-determination, Western Europe, Woodrow Wilson

## Abstract

The end of the First World War was a crucial time for nationalist leaders and minority communities across the European continent and beyond. The impact of the post-war spread of self-determination on the redrawing of Eastern European borders and on the claims of colonial independence movements has been extensively researched. By contrast, the international historiography has paid little attention to minority nationalist movements in Western Europe. This article focuses on three regions (Catalonia, Flanders and South Tyrol) that experienced considerable sub-state national mobilization in the interwar period. We aim to understand whether the leaders of Western European minorities and stateless nations shared the same enthusiasm as their anti-colonial and Eastern European counterparts for the new international order that self-determination seemed to foreshadow in the months following the end of the First World War. Because the American President Woodrow Wilson stood out as the most prominent purveyor of the new international legitimacy of self-determination, the article further examines how Western European nationalist movements exploited Wilson's image and advocacy to achieve their own goals. Nationalist forces in Catalonia, Flanders and South Tyrol initially mobilized self-determination and referred to Wilson as a symbol of national liberation, but this instrumentalization of self-determination was not sustained. Large-scale mobilization occurred only in Catalonia, and, even there, it disappeared suddenly in spring 1919. Furthermore, sub-state nationalist movements in Western Europe tended to mobilize self-determination to gain regional autonomy, rather than full independence, thus pursuing internal, not external, self-determination. The willingness of these movements to privilege autonomy over full independence made them more receptive to compromise. Radical forces would become stronger only in the 1930s and largely for reasons not directly connected to the post-war mobilization around self-determination.

## Introduction

The end of the First World War I was a crucial time for nationalist leaders and minority communities across the European continent and beyond. In Eastern Europe, the disintegration of the Austro-Hungarian Empire opened unexpected possibilities for the representatives of nationalities living under Habsburg rule to voice their claims for more autonomy or independence. In the colonial world, the rhetoric of self-determination promised to put an end to illegitimate imperial rule. In Western Europe, the immediate post-war months ushered in a time of nationalist contestation as well, despite the relative stability of state borders there.

The impact of the post-war spread of self-determination as a doctrine on the redrawing of Eastern European borders and on the claims of colonial independence movements has been researched extensively.^1^ However, international historiography has paid little attention to the nationalist movements of Western Europe that became active at the same time. Thus, how the increased international legitimacy of self-determination influenced the strategies of Western European nationalist movements in the immediate post-war months has largely remained unexplored, especially in a comparative perspective.^2^ This neglect betrays a persistent assumption that self-determination issues were, and are, not important in Western Europe, since this area is often supposed to be composed of homogenous nation-states.^3^

By contrast, during the interwar years several Western European regions displayed intense nationalist mobilization. Unfortunately, these events have mostly remained the domain of regional literatures that rarely speak to each other.^4^ A comparative historical perspective, taking into account simultaneous developments in several Western European areas, can draw together the insights available in these segregated historiographies and improve our knowledge of how self-determination was mobilized (or not) by local actors for their own specific purposes. This article carries out such comparative analysis by focusing on nationalist movements in Catalonia, Flanders and South Tyrol, three regions that went through processes of national mobilization throughout the interwar years. The main question that we answer is whether there was a similar mobilization of the language of self-determination in Western Europe as in the territories of the former Eastern European empires and in the colonial world.

The sense of opportunity on behalf of emerging nationalist leaders that was present in Europe and beyond towards the end of World War I stemmed, to a great extent, from a change in narrative emanating from the highest echelons of international politics. In January 1918, the American President Woodrow Wilson had publicized his 14 Point Programme, which laid out his vision of a post-war global order based on peace, justice and self-government for the nationalities of the Habsburg Empire. A month later, in his Four Points speech to the American Congress, the President openly embraced self-determination.^5^ Nationalist movements across the globe interpreted self-determination as an updated version of the older principle of nationalities that would legitimize their demands for full independence or regional autonomy within their state on the basis of their nation's ethnocultural characteristics.^6^

While Wilson’s role as a symbol of emancipatory struggles in the colonial world is well known (Erez Manela has most famously talked about a ‘Wilsonian Moment’),^7^ his influence on Western European nationalist movements is still unclear. Thus, we additionally examine how President Wilson's image and advocacy affected the narrative and actions of Western European regional leaders pursuing varying degrees of self-determination in the immediate post-war period. Examining Wilson's role does not mean minimizing the agency of local actors. On the contrary, we rather dissect how these actors exploited Wilson's advocacy in favour of self-determination to achieve their own goals.^8^ For this reason, we distance ourselves from the term ‘Wilsonian Moment’ and see the period in question as a time during which local leaders and movements developed a heightened awareness of the doctrine of self-determination and took advantage of its new international legitimacy.

Taking some distance from the expression ‘Wilsonian Moment’ is also warranted on account of the radicalizing impact of the First World War on nationalist movements throughout the continent and beyond, which constituted a key premise of the massive popularity acquired by self-determination from the end of 1918 onwards. Especially in continental empires, the extreme pressure of the war, coupled with a stalemate at the front and dramatic problems of food procurement, undermined imperial loyalties and opened a window of opportunity for nationalist politicians to mobilize populations along ethnic lines. Although nationhood was only one frame around which popular discontent could be organized, it did offer a powerful blueprint for new political projects out of the war impasse. This was also because in the last months of the war the sovereign claims of self-appointed representatives of imperial nationalities, either on the ground or in exile, increasingly met the official support of foreign powers.^9^ In this context, while it is certainly true that from late 1918 onwards Wilson's formula and personality gained centre stage and inspired many nationalist militants, the American President reaped a harvest that had been sown previously by other actors. Lenin had already defended self-determination in 1916. In November 1917, shortly after the Bolshevik Revolution, he turned this principle into official Russian policy and called for its application at the peace negotiations with the Central Powers that ended in the Treaty of Brest-Litovsk. Eyeing an opportunity to establish their influence on former Russian territories, German and Austro-Hungarian authorities agreed to place the principle at the core of the negotiations, albeit only for instrumental purposes. On 5 January 1918, David Lloyd George called for a settlement to the conflict based on the right to self-determination. Three days later, Wilson pronounced his famous 14 Point Programme in the American Congress and went on to become a global symbol of the rising star of self-determination, despite having been the last in a series of important statesmen to rally behind it.^10^

Nationalist forces in Western Europe did mobilize self-determination and refer to Wilson as a symbol of national liberation in ways similar to those used by movements in Eastern Europe and the colonial world. However, this instrumentalization of self-determination was not sustained. Large-scale mobilization in the name of self-determination did not occur in either Flanders or South Tyrol, and did not last in Catalonia. Furthermore, nationalist movements in Western Europe often mobilized self-determination to gain regional autonomy, rather than full independence. Finally, the willingness of Western European nationalist movements to keep autonomy as an option, rather than prioritizing full independence, made these movements receptive to compromise, both within their respective regional contexts and towards state authorities. Radical nationalist actors remained on the margins or quickly adopted approaches based on accommodation strategies. Extreme forces would become stronger only in the 1930s and largely for reasons not directly connected to the post-war mobilization around self-determination.

In the following, we will briefly review the concepts and approaches we use throughout the article. Then, we move on to discuss our case studies of Catalonia, Flanders and South Tyrol in detail.

## Comparing Self-Determination Demands in Western Europe: Some Preliminary Remarks

This article aims to understand whether the leaders of Western European minorities and stateless nations shared the same enthusiasm as their anti-colonial and Eastern European counterparts for the new international order that self-determination seemed to foreshadow in the immediate post-First World War period. The main questions that we answer are: firstly, in what ways did nationalist leaders in Western European regions harness the language of self-determination to advance their own calls for autonomy and/or independence; and secondly, in doing so, did they refer to President Wilson as a symbol of a new era of democracy and justice?

A second set of questions, which stem from Erez Manela's description of the evolution of self-determination demands in the colonial world, addresses the issue of whether nationalist movements radicalized after failing to reach their goals. In Manela's account, the nationalist euphoria that accompanied the spread of self-determination in the winter and spring of 1918–1919 ended in disillusion. Disappointment was such that anti-colonial leaders sought alternative programmes and found them in varied versions of an illiberal nationalism that amounted to a ‘revolt against the West’.^11^ Many anti-colonial leaders embraced radical socialism, while, a number of others, notably in the Arabic-speaking world, swung to the far right.^12^ Since in Western Europe calls for self-determination largely remained unheeded, the frustration of such requests begs the question whether we can identify short- or medium-term radicalization dynamics that are similar to those tracked by Manela. In the Western European context, we measure radicalization as a movement along two dimensions: an ideological drift towards the far right or the far left; and the rejection of the autonomist programmes that prevailed between late 1918 and mid-1919.

The prevalence of autonomism in the Western European context requires a clarification of our understanding of the concept of nationalism. In this article, we follow Ernest Gellner's assertion that nationalism is a political principle holding ‘that the political and the national unit should be congruent’,^13^ but we qualify it in one important respect. Gellner's definition implies that nationalism, especially stateless nationalism, is about achieving independent statehood. Although nationalism does tend to promote the pursuit of state power, we deem it reductive to attribute the nationalist label only to those organizations that seek external self-determination. The key goal of nationalist movements is sovereignty, not independent statehood as such, and sovereignty is compatible with forms of shared rule and participation in wider federal or confederal structures. As Walker Connor pointed out, nationalism is more about *choice* than about *result*, and, according to nationalist principles, ‘any nation has the right to secede, *if it so desires*’ (emphasis in the original).^14^ Hence, in this article, we consider movements as nationalist that other authors would define as regionalist on account of their demands for autonomy rather than independence.^15^ In contrast to most of the relevant historiography,^16^ we understand self-determination at the time in question to include *internal* self-determination (autonomy) and not just *external* self-determination (independence). To us, the decisive criterion for considering a movement as nationalist is the presence of a belief that a putative nation is a sovereign political community endowed with a right to decide about its political future, including sharing sovereignty with other political communities within a larger state structure. However, we are also aware that the line separating autonomism and secessionism is often very thin and political leaders can switch tactically from one to the other. This is a feature common to Catalonia, Flanders and South Tyrol, although throughout the early interwar period moderate and autonomist forces prevailed.

The advantage of a comparative approach is that it enables assessing historical trends in different places simultaneously. Such places, however, need to have some minimum features in common. Each of our case-study regions was inhabited by non-dominant groups with widespread feelings of belonging to a putative Catalan/Flemish/Tyrolean nation, although, in some cases, the precise contours and key features of the putative nation were still quite ill-defined, even among the elites. Furthermore, despite not constituting the generative moment of local nationalist movements, the immediate post-war months marked a critical juncture in the politicization of nationhood in all three locales under study. At the same time, in all three regions both nationalist politicians and the wider population tended to display an overlap between their nationalist beliefs and forms of allegiance to wider state structures and cultural aggregates (the Spanish and Belgian state for Catalan and Flemish moderate nationalists, the wider Dutch-speaking community for Flemish radicals, Austria and the German cultural area for South Tyrolean nationalists).

There are, however, also some notable differences. First, Catalonia and Flanders were home to two communities without a kin-state (although the Netherlands exercised some attraction for Flemish extremists) and did not experience any annexation to a new state as a consequence of the war. South Tyrol instead came under Italian control in 1918 and a substantial part of the region's population called for reunification with neighbouring North Tyrol (which was part of the new-born Republic of Austria). Second, Catalonia and South Tyrol were home to so-called high-status minorities (i.e., groups that were perceived as being endowed with considerable cultural, economic and/or political capital). The Flemish population, in contrast, represented a case of low-status majority, a feature that in the context of an expanding Belgian electoral franchise offered alternative options to regional autonomy that were not available to regional political elites in the other two cases. Third, although the First World War heightened the salience of ethnic and national differences in all of these three regions, in many other respects the impact of the conflict varied considerably in each of them. As Spain did not take part in the war, Catalonia was not directly harmed by the fighting, while the Great Powers had little interest in dealing with the domestic affairs of a neutral country. The war did contribute to further self-determination demands, but it also radicalized class conflicts that in the medium term hampered sub-state nationalist mobilization.^17^ In contrast, the restoration of Belgium was a declared war aim of the Entente and a cause that rallied the support of a majority of Belgians, although, paradoxically, the conflict simultaneously brought about the nationalist radicalization of a small, but influential, part of Flemish society.^18^ South Tyrol was the only one of these three regions that belonged to a defeated power and experienced annexation to a different state. Although historical records suggest that the region's fate was sealed fairly early in the Paris peace negotiations, this was not what external actors perceived. Therefore, a climate of uncertainty hovered over the future of the region until the summer of 1919.^19^ These differences notwithstanding, the three cases show similar patterns that help to advance our knowledge of trends in the mobilization of the language of self-determination in post-war Western Europe.

Finally, the article brings together regional historiographies that usually do not speak to each other and anchors them in the wider literature on self-determination and minority questions in interwar Europe and the colonial world. Furthermore, the article relies on both regional and state archives to investigate the local impact of the international diffusion of the principle of self-determination. Building on this material, in the next sections, we show how the months immediately after the end of the First World War generated a wave of unprecedented international legitimacy for self-determination claims that existing nationalist movements in Catalonia, Flanders and South Tyrol tried to ride in order to achieve their autonomist goals.

## Catalonia's Fleeting Campaign for Autonomy

In Catalonia, mobilization in favour of autonomy reached an unprecedented peak in late 1918 and early 1919. Explicitly referring to the doctrine of self-determination and celebrating President Wilson as a symbol of a new age of peace and justice, a wide movement for regional autonomy within the Spanish state grew within the existing Catalan political institutions and spilled over into the streets. The movement called for the recognition of Catalonia as a sovereign political community, but it refrained from demanding full independence. Although the movement embraced a wide section of the Catalan population, its political hegemony was undermined by the general indifference of the labour movement towards the campaign for autonomy. This created a structural weakness that jeopardized self-determination demands.

Catalan nationalism did not emerge at the end of the First World War, but rather originated in the mid-nineteenth century. In the first two decades of the twentieth century, different nationalist organizations arose, with ideological orientations ranging from the conservative right to the republican left. Most of them aimed at some form of recognition for the Catalan language and culture within Spain, but after the war the first separatist groups also appeared.^20^ By 1914, the *Lliga Regionalista* (Regionalist League), a conservative party having strong links with the Barcelona business community, had become the dominant political actor in the region. The *Lliga* promoted the idea that Catalonia was a separate nation from Spain and that the latter was a pluri-national state. More concretely, the party sought political autonomy for the four Catalan provinces of Barcelona, Gerona, Lerida and Tarragona. At the same time, aware that Catalonia was the most industrialized territory of the country, the *Lliga* also fancied becoming the leading force in the modernization of Spain. This second goal sometimes collided with the objective of Catalan self-government and several contemporary actors emphasized the *Lliga*'s ambivalent stand.^21^ Furthermore, by the end of the First World War Catalan nationalism had already turned into a mass movement (gathering between one third and one half of the seats for the Spanish Parliament allocated in the region), but it did not garner the support of the lower classes.^22^

In 1914, the *Lliga* had achieved the concession that the four Catalan provinces could join into a single administrative unit, called the *Mancomunitat*.^23^ However, the *Mancomunitat* did not receive any new competences from the central government. Hence, in 1917, several Catalan organizations joined the *Lliga* in an extra-parliamentary assembly in Barcelona that called for Catalan autonomy. A year later, after participating in two short-lived coalition governments without achieving these goals, the *Lliga*'s leadership, along with some Catalan Republicans, decided to seize the opportunity offered by the international hype around self-determination to launch a grassroots campaign for a regional statute of autonomy.^24^ An article published in the nationalist newspaper *La Veu de Catalunya* in October 1918 adequately conveyed the feeling of urgency shared within the *Lliga*. Under the title ‘Now or Never’, the piece encouraged fellow Catalans to realize that the world had changed. The world, the author continued, had declared that ‘peoples, big or small, must be allowed to enjoy all their freedom to the fullest’.^25^ Similar references to the changed international context and the coming of a new world in which peoples would be allowed to choose their future abounded in the nationalist press.

At about the same time, different Catalan actors began celebrating President Wilson as a hero for heralding a new era of peace and self-determination. The municipality of Barcelona made Wilson an honorary citizen of the city, a move that was later followed by several other Catalan town councils.^26^ Banquets were organized in the name of the American President, streets were renamed after him and the *Mancomunitat* proposed to grant him the title of ‘Friend and Guest of Catalonia’.^27^ At the end of October 1918, the internationalist Catalan monthly *Messidor* devoted its cover to Wilson, calling him a ‘symbol’ of ‘the aspiration of all noble and generous spirits … towards Justice, Truth and Goodness’.^28^ Part of this popularity was certainly due to the euphoria for the coming end of the war and was shared across Spain. France and the other Allied Powers were also celebrated in public commemorations along with Wilson. However, the American President did tend to attract a disproportionate amount of publicity and was strongly associated with pro-autonomy stances in Catalonia.^29^

The campaign for autonomy officially began in November 1918. At the end of that month, the *Mancomunitat* sent a proposal for self-government to the central executive. The document, which enjoyed widespread support as it had been approved by 98 per cent of Catalan municipalities, demanded ‘integral autonomy’ for the region, i.e., full sovereignty within specific devolved competences. In its conclusion, the text made a clear reference to the changed international context by referring to ‘this solemn moment in universal history, when the principle of the collective right of peoples to self-determination … is triumphant in the world’.^30^ During the parliamentary debate on the proposal, the majority of Spanish MPs strongly opposed the *Mancomunitat*'s far-reaching plan. As a consequence, the *Lliga*'s group in Parliament, along with the Catalan Republicans, left the assembly in protest.^31^

Polarization between supporters and opponents of Catalan autonomy spilled over into the streets. Anti-Catalan demonstrations spread throughout the country, while in Catalonia fights between Catalan and Spanish nationalist activists multiplied. At the end of December 1918, for instance, an editorial of the centrist newspaper *La Vanguardia* lamented the continuing riots caused by the ‘provocations’ of pro-autonomy militants and the brutal reaction of their opponents, a dynamic confirmed by the French consul in Barcelona in a report to Paris.^32^ The situation deteriorated in the following weeks and violence peaked on 26 January, when the *Mancomunitat* organized a public ceremony in Barcelona to present a draft Statute of Autonomy that would be submitted to the Spanish Congress. On the same day, clashes between members of the *Liga Patriotica Española* (LPE, Spanish Patriotic League) and the autonomy supporters who had flooded the Ramblas broke out and went on throughout the day, leading to several injuries and one death. The following day, in a stormy editorial on the *Correspondencia Militar*, the army threatened to kill all those Catalans who insulted Spain and her flag.^33^

Shortly after these events, Catalan MPs went back to Congress and tried to push through their proposal for autonomy. The debate dragged on until mid-February, when, for reasons unrelated to the campaign for self-government, the anarchist organization *Confederación Nacional del Trabajo* (CNT, National Confederation of Labour), the biggest trade union in Barcelona, called a major strike that paralyzed the city. The head of the incumbent liberal government, Prime Minister Romanones, closed down Parliament until a new election was held in June, thus ending parliamentary discussions on autonomy altogether.^34^ Facing mounting social unrest and accused of having contributed to exciting workers, the *Lliga* decided to put the autonomist campaign on the back burner and privilege the fight against the CNT.^35^ As the working classes did not show any concern regarding the end of the campaign, the rise of social protest killed the mobilization in favour of internal self-determination.^36^

The impact of the new international legitimacy of self-determination did not limit itself to the domestic constitutional debate. Catalan actors also endeavoured to reach out to President Wilson and to other foreign leaders directly. The *Lliga*'s MP Joan Ventosa went to Paris to lobby the Great Powers in December 1918, to the alarm of the French authorities, which even considered rejecting Ventosa's request for a visa to France.^37^ Further attempts at internationalizing the Catalan question came from the *Mancomunitat*, which sent copies of the Statute of Autonomy to all consuls in Barcelona, including those of Russia and the Austro-Hungarian Empire,^38^ as well as from small radical groups within the nationalist camp that addressed petitions to President Wilson and other diplomats.^39^ Although these attempts eventually failed, they worried Spanish diplomats and politicians, so much so that in a meeting with Wilson, in December 1918, Prime Minister Romanones sought reassurances from the American President that the Catalan question would not be considered at the Peace Conference.^40^

The above shows that between October 1918 and February 1919 the increased international legitimacy of self-determination generated considerable elite and popular mobilization in favour of Catalan autonomy. Yet, such enthusiasm for internal self-determination died away quite rapidly with the sudden rise of labour protests, and, although it did beget disappointment, neither the nationalist movement nor the wider population experienced substantial radicalization, at least not in the short term. For instance, in July 1919, the Council of the *Mancomunitat* sent a telegram to the President of the Peace Conference Clemenceau congratulating the Allies for the signature of the Versailles Treaty and welcoming the new age of ‘national and human liberty that it is now inaugurated’, despite the fact that the Catalan national cause had been utterly ignored in Paris.^41^ It is true that in March 1922 the *Lliga*'s youth branch formed a more radical party called *Acció Catalana* (Catalan Action). This however occurred as a consequence of another disappointing participation of the *Lliga* in government, rather than as a result of the failure of the campaign for autonomy in 1919. Radicalization did occur in the early 1930s, as embodied by the transition from the leadership of the autonomist *Lliga Regionalista* to that of *Esquerra Republicana de Catalunya* (Republican Left of Catalonia), a party that, although not openly separatist, did harbour stronger separatist tendencies and had a more far-reaching self-determination agenda. However, this radicalization mostly stemmed from the repression of Catalan nationalism, as well as the Catalan identity and language, pursued by the dictatorial government of General Miguel Primo de Rivera between 1923 and 1930.^42^ As argued by Borja de Riquer, ‘although the failure of the campaign for the statute [of autonomy] had radicalized some attitudes, many Catalanists still believed in the possibility of achieving autonomy within the monarchical parliamentary system’.^43^ Furthermore, although some members of the *Lliga* and of the Catalan bourgeoisie initially supported the dictatorship of General Primo de Rivera, this did not signal a shift towards the extreme right – and early support quickly turned into widespread criticism. Hence, in the years immediately following the failure of the campaign for autonomy, the Catalan nationalist movement did not slide towards more extreme ideological or separatist positions.

## Marginal Attempts at Self-Determination in Flanders

In the months following the end of the First World War, calls for Flanders’ autonomy within a Belgian federal state mostly concerned a relatively small, but influential, circle of extreme nationalist organizations and militants dominated by former soldiers and people who had worked with the German occupier. These also carried out transnational attempts to draw the attention of the Great Powers to the Flemish cause. As in Catalonia, such efforts failed completely. Contrary to the Catalan context, however, the mobilization of the language of self-determination did not concern moderate Flemish nationalists, which rather pushed for equality between French and Flemish within existing state structures. Unlike their Catalan counterparts, Flemish moderate nationalists could rely on Flanders’ demographic weight – the majority of Belgium's population lived in that region – to try to achieve their own linguistic and political goals within the existing Belgian political institutions and, thus, did not seek political autonomy.

Similar to Catalan nationalism, its Flemish counterpart arose well before the end of the Great War. Flemish nationalism originated in the second half of the nineteenth century as a cultural movement demanding linguistic equality between French and Flemish in a state dominated by francophone elites. At the turn of the twentieth century Flemish nationalism acquired a clearly political nature. Yet, before the Great War there was no explicit Flemish nationalist party and the nationalist movement (often simply called Flemish Movement) was an amalgam of different cultural organizations and political leaders campaigning for the linguistic emancipation of Flanders from within one of the three traditional parties.^44^

The First World War represented a fundamental watershed for the Flemish Movement in four major ways. First, playing on the contributions of the Flemish soldiers at the front, moderate nationalists, led by the Catholic politician Frans Van Cauwelaert, devised a ‘minimum programme’ calling for the full Dutchification of the administration, the courts and the educational system in Flanders, as well as for more linguistic equality between French and Dutch within the army. In 1919, this agenda commanded the support of about 40 per cent of MPs elected in Flanders and heavily influenced Belgian politics throughout the interwar years.^45^ Second, a small group of Flemish soldiers radicalized their Flemish nationalist ideals and founded the *Frontbeweging* (Front Movement), which later became the *Frontpartij* (FP, Front Party). The FP campaigned for Flemish sovereignty within a federal Belgium and was the first explicitly nationalist party in the history of Flanders.^46^ Third, some radical nationalists, then called activists, collaborated with German forces in occupied Belgium with the aim of implementing the Dutchification of Flanders. Immediately after the War, the activist legacy jeopardized the image of Flemish nationalism. In the long run, however, the activists managed to radicalize a substantial share of young Flemings, both in national and ideological (towards the extreme right) terms.^47^ Fourth, the First World War politicized ethnicity and nationhood to an unprecedented extent. This led to a paradoxical situation whereby the Great War marked the heyday of Belgian nationalism and, at the same time, saw the appearance of the first anti-Belgian programmes within the Flemish Movement.^48^

It is difficult to gauge the degree of identification of the Flemish-speaking population with Flemish nationalist goals and ideas. Maarten van Ginderachter has recently suggested that the earlier historiography has underestimated the strength of Flemish ethnicity among Flemish workers before the Great War. Yet, albeit pervasive, Flemish ethnicity was often not politicized and not in conflict with Belgian nationhood.^49^ Studies focusing on everyday nationalism provide substantial evidence that Flemish nationalism became a mass movement in the interwar years. Yet, this process was only at its inception in 1918–1919.^50^ Furthermore, separatist forces were marginal and moderate Flemish nationalist actors did not campaign for autonomy, but rallied behind the minimum programme.^51^ Most of the moderate Flemish Movement intended to take advantage of the demographic primacy of Flanders to make Belgium more Flemish and less francophone, rather than to obtain autonomy. At the same time, the ‘Dutchification’ of Flanders sought by the moderates amounted to a form of ‘cultural autonomy’ because it was supposed to insulate the region from the process of Frenchification that had occurred in the previous decades. Not accidentally, Frans Van Cauwelaert himself presented his minimum programme as ‘cultural autonomy with administrative adaptation’.^52^

Attitudes towards President Wilson were generally positive in Flanders, but moderate and radical forces made different uses of his image and legacy for their mobilization purposes. In the first half of 1919, the moderate nationalist newspaper *De Standaard*, founded by Van Cauwelaert, generally praised Wilson's idealism and defence of self-determination, sometimes bordering on hagiography.^53^ However, although one can find references to the liberation of the ‘small peoples’, the link between the Flemish struggle for linguistic emancipation and the doctrine of self-determination was not emphasized. By contrast, the newspaper *Ons Vaderland*, close to the FP, tried to capitalize more explicitly on Wilson's international advocacy. Until mid-1919, the paper displayed a cold, even critical, attitude towards the idealism of the US statesman. In the second half of that year, however, *Ons Vaderland* consistently celebrated Wilson as a pure idealist fighting for a world of peace and justice amidst corrupt and imperialist European leaders. The newspaper also openly stressed the relevance of Wilsonian self-determination for Flanders’ emancipation. For instance, in June 1919, the paper paid tribute to the American President, arguing that ‘we Flemings, who are a small and oppressed people, we nationalists, who fight for Flanders’ right to self-government, honour President Wilson as the great moral force in today's political society’.^54^ In September, an article defining Wilson as ‘the world judge’ advocated the inclusion of an article on the right of peoples to speak and be educated in their own language within the League's Covenant.^55^

In the Flemish context, the post-war higher legitimacy of self-determination mostly influenced the internationalization attempts of the activists and the *Frontpartij*, which however gathered the support of a minority in the region. The FP intended to send memorandums to Wilson and other statesmen at the beginning of 1919, but eventually did not realize these plans until the end of that year.^56^ In October, the party sent two open letters, one to President Wilson and the other to the Chairman of the Senate Committee of Foreign Relations, Henry Cabot Lodge. While the first mentioned the oppressed condition of the Flemish people,^57^ the second openly referred to the rights of the Flemish nationality to self-determination and asked Lodge to ‘use his mighty influence to secure autonomy for it within the Belgian state’.^58^ Probably suspecting that neither Wilson nor Lodge had read those letters, a month later, the FP sent some representatives to Paris to hand in a memorandum to the delegates still working at the Peace Conference. The document implored the Allied Powers to grant the Flemish ‘nationality’ the right to self-determination, while simultaneously reassuring foreign delegates that the party simply wanted Flanders to enjoy far-reaching autonomy within Belgium.^59^ This memorandum, however, did not have any influence on Belgian politics, whether it reached the Secretary of the Conference, Paul Dutasta, or not.^60^

The activists were quicker than FP members in their attempts to contact President Wilson. However, they were not more successful. In February 1919, the activists sent a message to the American President in which they asked him to consider the Flemings in the same way as the Eastern European nationalities that were being given a hearing at the Conference and to put pressure on the Belgian government to grant autonomy to Flanders.^61^ In April, the activists sent another memorandum to Wilson, but, once again, they only received an acknowledgment of receipt.^62^ We do not know whether Wilson saw either the memorandum or the previous messages sent by the activists. In any case, neither had any meaningful consequence for intergroup relations in Belgium.

Radicalization did occur in Flanders during the interwar years, but, as in Catalonia, it was linked to other domestic dynamics, notably the growing dissatisfaction of large swathes of the Flemish population with the linguistic reforms adopted in the early 1920s,^63^ rather than to any widespread disappointment with the failure of the rather marginal efforts to obtain internal self-determination in 1918–1919. This radicalization also mostly involved a new generation of young militants who had not participated in the attempts to lobby Wilson and other foreign leaders carried out in the immediate post-War months.^64^ *Ons Vaderland* continued to glorify the American President after the signature of the Treaty of Versailles, even if the Peace Conference completely ignored the ‘oppression’ of the Flemish people.^65^ By July 1919, *De Standaard* had grown more sceptical of the President's programme and action. Yet the paper mostly limited itself to abandoning the celebratory tone that it had previously used and to reporting news about Wilson in a neutral mode.^66^

Contrary to the Catalan case, in Flanders radical, not moderate, forces mobilized the language of self-determination and brandished Wilson as a symbol. Unlike their Catalan counterparts, Flemish moderate nationalists could pursue alternative strategies to try to obtain their goals of linguistic reform, different from regional autonomy. At the same time, in line with Catalan moderates, Flemish radicals did not call for independence, but rather limited their demands to the more modest goal of autonomy within the Belgian state. Furthermore, radical Flemish forces remained marginal within the Flemish Movement until the early 1930s and, when radicalization did occur, it was not driven by the dashing of hopes around self-determination of the immediate post-war months.

## South Tyrol: The Primacy of Unity with the North

While Catalonia and Flanders did not experience any regime change as a consequence of the First World War, South Tyrol was transferred from Habsburg to Italian rule. Although mass mobilization remained low, there was nearly total opposition to Italian annexation among Tyrolean elites. At the same time, there was no consensus over whether this meant joining the new-born Republic of Austria, or the German Reich, or declaring Tyrolean independence. What most Tyroleans agreed upon, however, was that North and South Tyrol had to remain united. Local and international actors discussed Tyrolean independence as an option throughout the end of 1918 and the first half of 1919, but often acknowledged its unrealistic nature. In this context, autonomy within a wider state entity (be it the Austrian Republic, the German Reich, or even Italy) was a much more likely outcome; one that mainstream political organizations in South Tyrol implicitly accepted when they began engaging in talks with Italian authorities over autonomy after the end of the Paris negotiations in the autumn of 1919. As in Catalonia and Flanders, disappointment concerning the annexation did not lead to immediate or mid-term radicalization. This appeared only in the early 1930s and depended more on a combination of fascist repression and Nazi success than on the failure of demands for self-determination in the immediate post-war months.

Already within the Austro-Hungarian Empire, Tyrolean society had developed a wide array of cultural, political and economic organizations aimed at sustaining the life of the regional community.^67^ With the transfer from the Empire to Italy, at the end of the Great War, most of these organizations became openly nationalist and defended South Tyrol's right to self-determination. The dominant political parties in the area during the Italian military occupation, which lasted until the end of summer 1919, were the *Tiroler Volkspartei* (Tyrolean People's Party), which was agrarian, conservative and Catholic, and *Die Freiheitliche Volkspartei* (the Liberal People's Party). In June 1919, these two merged into the *Deutscher Verband* (DV, German Association), which at the 1921 elections gathered 91 per cent of the votes cast in the region.^68^

Although it is difficult to know precisely the forms of identification that were prevalent among the wider Tyrolean population at the end of the War, broadly speaking, territorial allegiances moved between the following three foci of identification: Tyroleans saw themselves as members of the Crown Land of Tyrol, which entailed a sense of geographical unity, historical origin and common traditions; as members of Austria, which implied a politico-administrative sense of belonging to that half of the Empire (and later the Austrian Republic); and as members of the German cultural and linguistic area.^69^ These former Habsburg subjects did not necessarily identify strongly as members of the Austrian Republic or the German Reich, but, as Roberta Pergher has argued, ‘they did recognize the need in this new “postimperial” world to belong to a nation-state’ and ‘they found themselves in a nation that was clearly not their “natural” home’.^70^ Above all, the priority for most Tyroleans in the early post-war period was keeping North and South Tyrol united. As a member of the British legation to Vienna reported from a trip to Tyrol in February 1921, ‘what struck me in these conversations was that my interlocutors all took the Tyrolese point of view for granted. I should say they were all thinking of Tyrol first’.^71^

Initially, most of the German-speaking elite in South Tyrol simply thought that Wilson's 14 Points would guarantee the region's unity with North Tyrol in accordance with the right of self-determination.^72^ Not believing that the Italian military authorities could annex the region, South Tyrolean leaders did not see the need to collaborate with them and some even completely rejected the Italian occupation. On 4 November 1918 (before the Italian army even entered the city), the mayor of Bolzano/Bozen Julius Perathoner and other local leaders drafted a manifesto for self-determination and set up a South Tyrolean Provisional National Assembly, which existed until January 1919. Around the same period, the mayor of the city of Merano/Meran, Count Marzani, declared his loyalty to Austria and was followed by the members of his administration – for this, he was later expelled to Austria by the Italian military.^73^

Para-diplomatic efforts accompanied these open acts of defiance of the Italian occupation. Both North and South Tyrolean politicians tried to lobby international actors to avoid the division of Tyrol. To this effect, its members proposed different territorial options to the Great Powers and to neighbouring countries (an independent state, an autonomous demilitarized region in Austria and, even, an autonomous region in Italy).^74^ Between late 1918 and early 1919, Tyrolean delegates tried to approach leaders of the US, France and the UK, especially in Switzerland. In March/April 1919, the South Tyrolean politician Paul von Sternbach travelled to Berne to hand a memorandum to Wilson asking the American President to recognize South Tyrol's self-determination and oppose its incorporation into Italy. Signed by all mayors of the province, the document could not be given directly to Wilson, and was therefore left at the American delegation in the Swiss capital. Copies were also distributed to the French, British, German and Austrian embassies in the city.^75^ A similar memorandum was also sent to the Great Powers at the Peace Conference at the end of March 1919. The document defined South Tyrol as ‘the heart of Tyrol and the cradle of our race’ and asserted that ‘today, when President Wilson and together with him the Peace Conference guarantee the free self-disposition [sic] of peoples as the basis of Peace, it becomes our proper right also to claim for ourselves what is granted to for [sic] others’.^76^ We do not know whether Wilson received these and other memorandums that Tyrolean politicians sent to him in those early months of 1919, but what is certain is that they did not manage to influence the American President as these politicians wished.

In his reports to fellow nationalists in Tyrol, von Sternbach stressed how the members of several foreign delegations, albeit always unofficially and never on behalf of their countries, advised the Tyroleans to declare independence in order to put pressure on the negotiators in Paris.^77^ The idea of a demilitarized, neutral, independent Tyrol, possibly including Voralberg, Salzburg and parts of Carinthia and Styria too was being shared also within British diplomatic circles. The constitution of such a ‘second Switzerland’ had the undoubted advantage of potentially being able to assuage Italian strategic concerns, while at the same time respecting the principle of self-determination to a greater degree than the Italian annexation of South Tyrol. Given the extremely uncertain existence of the Austrian Republic, it was deemed not to be such a far-fetched scenario.^78^ In South Tyrol, the Bolzano/Bozen branch of the *Tiroler Volkspartei* had already floated the idea of declaring Tyrol an independent country at a session of the Provisional Assembly of Tyrol in January 1919.^79^ Yet there was scepticism about external self-determination. At a meeting of Tyrolean delegates in Innsbruck in March of the same year, the majority of the assembly preferred to wait and see what would happen in Paris before declaring an independence that would be difficult to sustain given the small size of the area.^80^ In May 1919, the North Tyrolean Assembly issued a declaration in which it warned that it would declare the independence of the entire region if this was the only way to ensure its territorial integrity, adding that, if Tyrol ended up divided, the North would join Germany.^81^

The independence option was still raised on a few occasions after the adoption of the Treaty of St. Germain. Yet the old concerns about the viability of an independent Tyrol continued to undermine it.^82^ An alternative consisted in the constitution of an autonomous unified Tyrol within Italy. Members of the Tyrolean Diet in Innsbruck (at the time still under Italian occupation) supposedly made this proposal to the Italian Captain Pietro Stoppani in the summer of 1919. These Tyrolean political leaders asked the Italian officer to report to the King their offer to join Italy as a unified country, so that they could avoid the split of the South from the North.^83^

However, Tyrolean para-diplomatic efforts failed. By the autumn of 1919, South Tyrolean political leaders understood that their annexation by Italy was irreversible and the DV began engaging with Rome in negotiations for autonomy.^84^ Yet, no deal could be struck before the Italian fascists took over in October 1922 and began implementing their own assimilationist plans for South Tyrol. Disappointment had already started growing in spring 1919 and in the local press President Wilson became a prime target of Tyrolean anger. In April, the local nationalist paper *Der Tiroler* began criticizing Wilson for the inconsistent application of his programme. Because of press censorship, the paper vented its frustration in reporting about the situation of other nations across the globe that had believed in the American leader and had been betrayed by him.^85^ At the end of 1919, an article lambasted Wilson as a Messiah who had disappointed all the people who had believed in his programme, while the three Great Powers (the US, England and France) had profited from the peace to consolidate their empires.^86^

Disappointment flared up again in October 1920, when the Italian Parliament adopted a law sanctioning the annexation of South Tyrol. A few days later *Der Tiroler* defined the Versailles Peace as a ‘violent peace’ and Wilson as the main politician responsible for it.^87^ Ten days later, the *Andreas Hofer Bund* – a nationalist organization founded in Innsbruck in the summer of 1919 – sent an open letter to the American President in which it called the annexation ‘one of the most shameful sell-outs that secret diplomacy has ever contrived’.^88^

Although the confirmation of the Italian annexation of South Tyrol frustrated regional political actors, these continued to abide by a moderate policy of cooperation, mostly aiming at obtaining autonomy during the liberal period and trying to preserve the cultural and linguistic features of the area as much as possible under Fascism – essentially by means of secret institutions.^89^ Radicalization occurred only from 1933 onwards, when National Socialism began spreading among the South Tyrolean population and had as its main promoter the *Völkischer Kampfring Südtirol* (VKS, People's Combat Alliance of South Tyrol). The rise of the VKS also went along with a general reorientation of the local German-speaking population away from the tutelage of Austrian authorities and ever more towards the sphere of influence of the *Reich*, which occurred after the Referendum on the Saar in 1935. However, such radicalization depended on a decade of oppressive dictatorship and the international strengthening of extreme right and Greater German ideals, rather than on the failed mobilization around self-determination of the immediate months after the First World War.^90^

## Conclusion: A Collage of Local Adaptations

Studies of the post-war spread of self-determination have tended to stress the popular strength of nationalist advocacy and focused on leaders and organizations campaigning for full independence. By contrast, the previous sections have shown that mobilization in favour of self-determination in Catalonia, Flanders and South Tyrol remained mostly limited to political elites. Furthermore, nationalist movements chiefly sought autonomy, rather than independence, and were often willing to seek compromise with state authorities.^91^ While radical forces did exist, they tended to be marginal and moderate leaders held the reins of power within their respective movements well into the late 1920s.

The greater international legitimacy that the principle of self-determination acquired in 1918–1919 offered a window of opportunity to already existing nationalist actors to pursue their political goals. However, these actors rarely managed to draw the masses into their protest action. When a degree of mass mobilization occurred, as during the campaign for autonomy in Catalonia, it was partial (since it did not include most of the working class) and ephemeral (as it evaporated almost overnight with the rise of social protest). Furthermore, while the international spread of the doctrine of self-determination in the immediate post-war months did function as a catalyst for local political contestation, this does not imply that radical voices won out. Moderate leaders and parties (even in ideological terms) prevailed throughout the immediate post-war months and generally followed instrumental policies aimed at finding compromise solutions. Nationalist actors generally sacrificed matters of principle to pragmatic strategies. This was the case for instance with the campaign for autonomy in Catalonia, where the *Lliga Regionalista* favoured the fight against the trade unions over the pursuit of self-government.

Accordingly, demands for self-determination in Western Europe after the First World War mostly took an autonomist form. Nationalist actors in Catalonia and Flanders did not threaten territorial integrity and were rather satisfied with obtaining extensive regional powers within existing state structures. In South Tyrol, where the option of independence was continuously voiced between late 1918 and the summer of 1919, nationalist forces also recognized its unrealistic nature. Autonomy (be it within Austria, Germany, or Italy) thus emerged as the most likely outcome of any diplomatic negotiations and when the Peace Conference confirmed Italian annexation, mainstream South Tyrolean leaders began discussing autonomy with Italian authorities in earnest.

None of the movements that tried to exploit the heightened awareness of self-determination in the immediate post-war months in Catalonia, Flanders and South Tyrol reached its goals. The front led by the *Lliga Regionalista* did not obtain autonomy, nor did the FP or the activists, while the wider Flemish Movement, which had not mobilized the language of self-determination, won only piecemeal linguistic reform. The DV did not manage to keep North and South Tyrol together or to conclude negotiations for regional autonomy before the rise of Fascism. The failure of these movements generated widespread discontent. It did not beget immediate, or even medium-term, radicalization. In all three areas, less compromising leaders and political parties only became popular later in the interwar years, notably during the 1930s. It is difficult to consider this a direct legacy of the dashed hopes of late 1918 and early 1919. Several other factors account for a radicalization that took about a decade to build up and often involved younger leaders who had not taken part in the events of the immediate post-war months. In Italy and Spain, the consequences of the repressive policies enacted by the dictatorships of Benito Mussolini and General Miguel Primo de Rivera dwarf the impact of the failure of the post-war hype around self-determination on the growth of *Esquerra Republicana de Catalunya* and the *Völkischer Kampfring Südtirol* respectively, as well as, more generally, on wider popular support for self-determination demands. In Belgium, prolonged resistance against linguistic reform in Flanders played the biggest role in boosting the electoral chances of anti-Belgian nationalism.

Above all, the impact of the broader international legitimacy of self-determination in Catalonia, Flanders and South Tyrol was not a uniform phenomenon, but rather a collage of local versions of partially successful, partially aborted, attempts to ride a wave of unprecedented international support for minority nationalist causes that, however, happened to be more fleeting than nationalist leaders could have hoped for.

## Notes

1 Among others see: Alan Sharp, ‘The Genie That Would Not Go Back into the Bottle: National Self-Determination and the Legacy of the First World War and the Peace Settlement’, in Seamus Dunn and T. G. Fraser, eds, *Europe and Ethnicity: World War I and Contemporary Ethnic Conflict* (London 1996), 10–29; Margaret Macmillan, *Paris 1919: Six Months That Changed the World* (New York 2001); Erez Manela, *The Wilsonian Moment: Self-Determination and the International Origins of Anticolonial Nationalism* (Oxford 2007); Sarah Claire Dunstan, ‘Conflicts of Interest: The 1919 Pan-African Congress and the Wilsonian Moment’, *Callaloo*, Vol. 39, No. 1 (2016), 133–50; Michael Goebel, *Anti-Imperial Metropolis: Interwar Paris and the Seeds of Third World Nationalism* (New York 2017).

2 For exceptions concerning Catalonia, Ireland and France see: Xosé Manoel Núñez Seixas, *Internacionalitzant el nacionalisme: el catalanisme polític i la qüestió de les minories nacionals a Europa (1914–1936)* (València 2010); Martin O’Donoghue, ‘“Ireland’s Independence Day”: The 1918 Election Campaign in Ireland and the Wilsonian Moment’, *European Review of History: Revue Européenne d’histoire*, Vol. 26, No. 5 (2019), 834–54; Francesca Zantedeschi, ‘Micro-Nationalisms in Western Europe in the Wake of the First World War’, in Xosé Manoel Núñez Seixas, ed., *The First World War and the Nationality Question in Europe: Global Impact and Local Dynamics* (Leiden 2020), 145–69. For some comparative conclusions see: Eric Storm and Maarten Van Ginderachter, ‘Questioning the Wilsonian Moment: The Role of Ethnicity and Nationalism in the Dissolution of European Empires from the Belle Époque through the First World War’, *European Review of History: Revue Européenne d’histoire*, Vol. 26, No. 5 (2019), 747–56; Xosé Manoel Núñez Seixas, ‘Wilson’s Unexpected Friends: The Transnational Impact of the First World War on Western European Nationalist Movements’, in Xosé Manoel Núñez Seixas, ed., *The First World War and the Nationality Question in Europe: Global Impact and Local Dynamics* (Leiden 2020), 1–16. For a comparative study on sub-state nationalist and regionalist movements in Europe in the nineteenth century see Joost Augusteijn and Eric Storm, eds, *Region and State in Nineteenth-Century Europe: Nation-Building, Regional Identities and Separatism* (Basingstoke 2012). For a longer historical perspective that also includes the interwar period see Xosé Manoel Núñez Seixas and Eric Storm, eds, *Regionalism and Modern Europe: Identity Construction and Movements from 1890 to the Present* (London 2019).

3 John Coakley, ‘Reifying Ethnicity? Measuring National Identity in Europe’ (APSA 2011 Annual Meeting, Rochester, NY, 2011).

4 For exceptions, see note 2 above.

5 Eric D. Weitz, ‘From the Vienna to the Paris System: International Politics and the Entangled Histories of Human Rights, Forced Deportations, and Civilizing Missions’, *The American Historical Review*, Vol. 113, No. 5 (2008), 1328.

6 Weitz, ‘From Vienna’, 1328–29; Núñez Seixas, ‘Wilson’s Unexpected’, 49–50.

7 Manela, *The Wilsonian Moment*, 6–7.

8 Minimizing the agency of local nationalist actors is a criticism that has been made against Manela. See Rebecca E. Karl, ‘Review of *The Wilsonian Moment: Self-Determination and the International Origins of Anticolonial Nationalism*’, *The American Historical Review*, Vol. 113, No. 5 (2008), 1474–76.

9 Aviel Roshwald, *Ethnic Nationalism and the Fall of Empires: Central Europe, the Middle East and Russia* (London 2000), 70–115; Jörn Leonhard, *Pandora’s Box: A History of the First World War* (Cambridge, MA 2018), 799–817; Robert Gerwarth, *The Vanquished: Why the First World War Failed to End, 1917–1923* (London 2017), 385–411.

10 Derek Benjamin Heater, *National Self-Determination: Woodrow Wilson and His Legacy* (New York 1994), 36–37; Borislav Chernev, ‘The Brest-Litovsk Moment: Self-Determination Discourse in Eastern Europe before Wilsonianism’, *Diplomacy & Statecraft*, Vol. 22, No. 3 (2011), 369–87.

11 Manela, *The Wilsonian Moment*, 224.

12 Goebel, *Anti-Imperial*, 176–217, 269–78.

13 Ernest Gellner, *Nations and Nationalism* (Ithaca, NY 1983), 1. While Gellner’s *theory* of nationalism as a consequence of industrialization has been challenged on both theoretical and empirical grounds, his *definition* of nationalism is still widely used. Our use of Gellner’s definition, qualified on the basis of insights from Walker Connor’s work, does not imply that we subscribe to his explanation of nationalism. For a critical overview of traditional approaches to the study of nationalism see Umut Özkirimli, *Theories of Nationalism: A Critical Introduction* (London 2017). For important recent contributions see Andreas Wimmer, *Nationalist Exclusion and Ethnic Conflict: Shadows of Modernity* (New York 2002); Rogers Brubaker, *Ethnicity without Groups* (Cambridge 2004); Aviel Roshwald, *The Endurance of Nationalism: Ancient Roots and Modern Dilemmas* (Cambridge 2006); Rogers Brubaker, *Nationalist Politics and Everyday Ethnicity in a Transylvanian Town* (Princeton, NJ 2006); Michael Skey, *National Belonging and Everyday Life: The Significance of Nationhood in an Uncertain World* (Basingstoke 2011); Bernard Yack, *Nationalism and the Moral Psychology of Community* (Chicago, IL 2012); Andreas Wimmer, *Nation Building: Why Some Countries Come Together While Others Fall Apart* (Princeton, NJ 2020).

14 Walker Connor, ‘Democracy and National Self-Determination: Yesterday, Today, and Tomorrow’, in Begoña Aretxaga et al., eds, *Empire and Terror: Nationalism/Postnationalism in the New Millennium* (Reno, NV 2005), 23.

15 For a broader discussion of the differences between regionalism and nationalism see Xosé Manoel Nuñez Seixas and Eric Storm, ‘Introduction: Region, Nation and History’, in Nuñez Seixas and Storm, eds, *Regionalism and Modern Europe*, 1–23.

16 See for instance Manela, *The Wilsonian Moment*; Storm and Ginderachter, ‘Questioning’; Nuñez Seixas, ‘Wilson’s Unexpected’.

17 Francisco Romero Salvadó and Angel Smith, ‘The Agony of Spanish Liberalism and the Origins of Dictatorship: A European Framework’, in Francisco J. Romero Salvadó and Angel Smith, eds, *The Agony of Spanish Liberalism: From Revolution to Dictatorship 1913–23* (Basingstoke 2010), 13–17.

18 Jean Stengers and Éliane Gubin, *Le grand siècle de la nationalité belge: de 1830 à 1918* (Bruxelles 2002), 186–8.

19 Macmillan, *Paris 1919*, 290–1; Andrea Di Michele, *L’italianizzazione imperfetta: l’amministrazione pubblica dell’Alto Adige tra Italia liberale e fascismo* (Alessandria 2003), 50–9.

20 On Catalan nationalism before the First World War see: Albert Balcells, *El nacionalismo catalán* (Madrid 1991); Borja de Riquer i Permanyer, *Escolta, Espanya: la cuestión catalana en la época liberal* (Madrid 2001); Enric Ucelay da Cal, *El imperialismo catalán: Prat de la Riba, Cambó, D’Ors y la conquista moral de España* (Barcelona 2003).

21 Borja de Riquer i Permanyer, ‘Persistencia y cambios en la Cataluña de la Restauración’, *Anales de la Universidad de Alicante: Historia contemporánea*, No. 7 (1989), 129–30.

22 Xosé M. Núñez Seixas, ‘Catalonia and the “War of Nations”: Catalan Nationalism and the First World War’, *Journal of Modern European History*, Vol. 16, No. 3 (2018), 381–6; Angel Smith, ‘The Lliga Regionalista, the Catalan Right and the Making of Primo de Rivera Dictatorship, 1916–1923’, in Francisco J. Romero Salvadó and Angel Smith, eds, *The Agony of Spanish Liberalism: From Revolution to Dictatorship 1913–23* (Basingstoke 2010), 157–65.

23 Francisco J. Romero Salvadó, ‘Between the Catalan Quagmire and the Red Spectre, Spain, November 1918–April 1919’, *The Historical Journal*, Vol. 60, No. 3 (2017), 800.

24 Charles E. Ehrlich, ‘Per Catalunya i l’Espanya Gran: Catalan Regionalism on the Offensive, 1911–19’, *European History Quarterly*, Vol. 28, No. 2 (1998), 208–10; Javier Moreno Luzón, ‘De agravios, pactos y símbolos. El nacionalismo español ante la autonomía de Cataluña (1918–1919)’, *Ayer*, No. 63 (2006), 121–2.

25 Josep Bertran i Musitu, ‘Ara o mai’, *La Veu de Catalunya*, 14 October 1918, evening issue, 8. See also, ‘La urgencia nacionalista’, *La Veu de Catalunya*, 17 October 1918, evening issue, 8.

26 ‘El President Wilson, barceloni honorari’, *La Veu de Catalunya*, 17 October 1918, evening issue, 8.

27 Document without date entitled ‘Honorable Senyor’ in Arxiu Nacional de Catalunya (ANC), 737–5489; ‘Generalitat’, *La Veu de Catalunya*, 19 December 1918, evening issue, 7. It is not clear whether the invitation was ever sent though.

28 Paul Turull, ‘Wilson, simbolo’, *Messidor*, 29 October 1918, 203. See also, ‘Recorda’t!’, *La Trinxera Catalana*, 19 December 1918, 1, quoted in Núñez Seixas, ‘Catalonia’, 392.

29 Enric Ucelay-Da Cal, ‘Wilson i no Lenin: L’esquerra catalana i l’any 1917’, *L’Avenç*, No. 9 (1982), 53–8; Guillermo Pérez Casanova, ‘!Sálvanos, Wilson!: El remedio americano para la decadencia europea’, in *Coetánea: III Congreso Internacional de Historia de Nuestro Tiempo* (2012), 187–96.

30 Consell de la Mancomunitat de Catalunya, *Bases per l’autonomia de Catalunya*, 25 November 1918, ANC, 1-737-5374. Copies of the proposal (in Spanish and other languages) are to be found at ANC, 1-737-5373. The demand for autonomy was supported by hundreds of associations from throughout Catalonia, see ANC, 1-737-5351.

31 Congreso de los diputados, *Diario de las sesiones de Cortes*, No. 107, 12 December 1918, 3518.

32 Moreno Luzón, ‘De agravios’, 128–30. ‘Los alborotos callejeros’, *La Vanguardia*, 24 December 1918, 7. Consulate in Barcelona to embassy in Madrid, 29 January 1919, Centre des Archives diplomatiques de Nantes (CADN), 72PO/2/52.

33 ‘Los succesos de la Rambla’, *La Vanguardia*, 27 January 1919, 6; ‘Aclarando y sosteniendo nuestra actitud’, *La Correspondencia Militar*, 27 January 1919, 1; see also documents in ANC, 1-737-5427.

34 Salvadó, ‘Between the Catalan’, 805–11; Borja de Riquer i Permaner, *Alfonso XIII y Cambó. La monarquía y el catalanismo político* (Barcelona 2013), 126–31.

35 Smith, ‘The Lliga’, 155.

36 On Catalan nationalism among industrial workers see José Luis Oyón, ‘Anarquismo y antinacionalismo en Solidaridad Obrera, 1930–1936’, in José Luis Oyón and Juanjo Romero Marín, eds, *Clase antes que nación: trabajadores, movimiento obrero y cuestión nacional en la Cataluña metropolitana, 1840–2017* (Vilassar de Dalt 2017), 361–429, 371. See also, the contemporary comments of Raimond Negre i Balet in ‘Catalunya devant la propera pau’, *Messidor*, 29 October 1918, 214–16.

37 Exchange between the Minister of Foreign Affairs Pichon, the French Ambassador in Madrid and the Consul in Barcelona of 14 and 15 December 1918 in CADN, 396PO-C-139. Unfortunately, there is no mention of this trip in the rich correspondence between Cambo and Ventosa. Biblioteca de Catalunya (BC), Fondo Borras, Correspondencia Cambo-Ventosa, Folder C2b.

38 ANC, 1-737-5485.

39 Facsimil del missatge qu’els nacionalistes catalans han endreçat al capitost de la democracia del Segle XX, Mr. Wilson, ANC, 737-5484; Núñez Seixas, *Internacionalitzant*, 75–82.

40 Núñez Seixas, *Internacionalitzant*, 75–82; Archivo Histórico Nacional (AHN), Ministerio de Asuntos Exteriores (MAE), H3054, Ex. 21.

41 ANC, 1-737-5106.

42 José Luis de la Granja Sainz, Justo G. Beramendi, and Pere Anguera, *La España de los nacionalismos y las autonomías* (Madrid 2001), 113.

43 de Riquer, *Alfonso XIII*, 132.

44 Lode Wils, ‘Het Ontstaan van de “Kleine” Vlaamse Natie’, *Wetenschappelijke Tijdingen*, Vol. 50, No. 4 (1991), 220–38; Romain Vanlandschoot, ‘Sociale Beweging En Vlaamse Beweging’, *Wetenschappelijke Tijdingen* Vol. 52, No. bijzonder (1993), 43–57.

45 Lode Wils, *Onverfranst, onverduitst? Flamenpolitik, activisme, frontbeweging* (Kalmthout 2014), 312.

46 Luc Vandeweyer, ‘“Een Ruim Veld Voor de Studenten-Beweging Ligt Open”. Inzake Oorsprong En Aard van de Vlaamse “Frontbeweging”’, *Wetenschappelijke Tijdingen* Vol. 77, No. 3 (2018), 213–30; Luc Vandeweyer, ‘Robrecht De Smet in Het Vlaamse Front 1918–1920’, *Wetenschappelijke Tijdingen*, Vol 43, No. 3 (1984), 173–85.

47 Louis Vos, ‘De Politieke Kleur van Jonge Generaties. Vlaams-Nationalisme, Nieuwe Orde En Extreem-Rechts’, in Rudi van Doorslaer, ed., *Herfsttij van de 20ste Eeuw: Extreem-Rechts in Vlaanderen 1920–1990* (Leuven 1992), 312–68.

48 Stengers and Gubin, *Le grand siècle*, 186–88.

49 Maarten Van Ginderachter, ‘How to Gauge Banal Nationalism and National Indifference in the Past: Proletarian Tweets in Belgium’s Belle Epoque’, *Nations and Nationalism*, Vol. 24, No. 3 (2018), 579–93.

50 Anne Himpe, *De beweging in huis: Vlaamse huizen tijdens het interbellum* (Gent, 1992), 12–44; Barbara Deruytter, ‘The Layering of Belgian National Identities during the First World War’, in Nico Wouters and Laurence Van Ypersele, eds, *Nations, Identities and the First World War: Shifting Loyalties to the Fatherland* (London 2018), 155–74, 168; Koenraad De Meulder and Jan Dewilde, ‘Het lied in ziel en mond’, in Guy Leemans et al., eds, *Vlamingen komt in massa: de Vlaamse beweging als massabeweging* (Gent 1999), 199–226; Bruno De Wever, *Greep Naar de Macht : Vlaams-Nationalisme En Nieuwe Orde: Het VNV 1933-1945* (Tielt 1994), 61–4.

51 Wils, *Onverfranst*, 301.

52 Ibid. Concerning the link between the moderate nationalist faction led by Van Cauwelaert and the activists, Bruno De Wever concludes that the dispute between the moderate Flemish movement and moderate activists was not a fundamental one, but simply one of method: both wanted the Flemish interest to prevail over the Belgian one. De Wever, *Greep*, 26.

53 ‘International Overzicht. Wilson’, *De Standaard,* 28 April 1919, 1. See also, ‘Het leven van Wilson’, *De Standaard*, 28 April 1919, 1; ‘Hulde aan President Wilson’, *De Standaard*, 18 June 1919, 1.

54 ‘Heil Wilson’, *Ons Vaderland*, 19 June 1919, 1. See also, ‘Ondank is ‘s werelds loon’, *Ons Vaderland*, 20 June 1919, 1; ‘Na het bezoek van Wilson’, *Ons Vaderland,* 21 June 1919, 1; ‘Hooggeachte Heer’, *Ons Vaderland*, 1 August 1919, 1.

55 ‘Het Taalrecht en de Vrijheid der Volken’, *Ons Vaderland,* 26 September 1919, 1.

56 Vandeweyer, ‘Robrecht de Smet’.

57 Brief aan Woodrow Wilson, 20 October 1919, in Guy Leemans et al., eds, *‘Alleen in u – o koning – geloven wij nog’: open brieven van de Vlaamse Frontbeweging tijdens de Eerste Wereldoorlog* (Antwerp 2017), 327–9.

58 Brief aan Senator Lodge, 20 October 1919, in Leemans et al., *‘Alleen’*, 335.

59 ‘Aux Délégués des peuples à la Conférence de la Paix’, Brussels, 20 November 1919, Diplomatiek archief van België/Archives diplomatiques de Belgique (DAB/ADB), 257, dossier général 1913–1934.

60 ‘Lettre de Paris. La Délégation du Vlaamsche Front’, translation from the Ministry of Foreign Affairs of an article that appeared in *Ons Vaderland* on 7 December 1919, DAB/ADB, folder 257, dossier général 1913–1934; ‘Une bombe raté’, *La Nation Belge*, 5 December 1919, in the above folder.

61 Fallon to the Ministry of Foreign Affairs, 5 March 1919; Belgian Army, Commandment of military security to the Minister of Foreign Affairs, 2 May 1919. Both documents are in DAB/ADB, 257 dossier général 1913–1934.

62 Flemish Committee, *Pro Flandria Servanda: Flanders’ Right and Claim for Autonomy, Formulated, Explained, Justified* (The Hague 1920), 86–7.

63 Emmanuel Gerard, ‘La démocratie rêvée, bridée et bafouée (1918–1939)’, in *Nouvelle Histoire de Belgique: 1905–1950*, Michel Dumoulin et al., eds., vol. 2 (Bruxelles 2006), 172–87.

64 Vos, ‘De Politieke’; De Wever, *Greep*, 70–82.

65 ‘Het Taalrecht en de Vrijheid der Volken’, *Ons Vaderland*, 26 September 1919, 1.

66 See for instance ‘De openbare meening en President Wilson’, *De Standaard*, 14 January 1920; ‘Uittreiking van de Nobel-prijs voor de Vrede’, *De Standaard*, 12 December 1920, 1.

67 Laurence Cole, ‘Province and Patriotism: German Cultural Identity in Tirol in the Years 1850–1914’, *Österreichische Zeitschrift Für Geschichtswissenschaften*, No. 6 (1995), 61–83.

68 Karl Trafojer, ‘Die innenpolitische Lage in Südtirol: 1918–1925’ (Wien 1971), 166–8. Michael Gehler, *Eduard Reut-Nicolussi und die Südtirolfrage 1918–1958: Streiter für die Freiheit und die Einheit Tirols*, vol. 1 (Innsbruck 2007), 34–6.

69 Leopold Steurer, *Südtirol zwischen Rom und Berlin, 1919–1939* (Wien 1980), 37.

70 Roberta Pergher, *Mussolini’s Nation-Empire: Sovereignty and Settlement in Italy’s Borderlands, 1922–1943* (Cambridge 2018), 56.

71 Lindley to Curzon, Situation in Salzburg and Tyrol, 3 February 1921, C 2657/2657/3, The National Archives (TNA), Foreign Office (FO) 371-5780.

72 Claus Gatterer, *Im Kampf gegen Rom: Bürger, Minderheiten und Autonomien in Italien* (Wien 1968), 289–305; Rolf Steininger, *Südtirol im 20. Jahrhundert: Vom Leben und Überleben einer Minderheit* (Innsbruck 2016), 29–38.

73 Di Michele, *L’italianizzazione imperfetta*, 47.

74 Steininger, *Südtirol*, 29–38. On the para-diplomatic activities of Tyrolean nationalist politicians see also Marion Dotter and Stefan Wedrac, *Der hohe Preis des Friedens: die Geschichte der Teilung Tirols, 1918-1922* (Bozen/Innsbruck 2018), 98-137; Oswald Überegger, *Im Schatten des Krieges: Geschichte Tirols 1918-1920* (Paderborn 2019), 77-136.

75 For the memorandum see Denkschrift Deutschsüdtirols, Februar 1919, in Michael Gehler and Eduard Reut-Nicolussi, *Eduard Reut-Nicolussi und die Südtirolfrage 1918–1958: Streiter für die Freiheit und die Einheit Tirols. Dokumentenedition*, vol. 2 (Innsbruck 2007), 34–40.

76 Rumbold to Curzon, Italian Claims in South Tyrol, 26 March 1919, dispatch n. 180, p. 474, TNA, FO 608-15.

77 Report of Prof. Heidegger and von Sternbach about their travels to Switzerland, no date, Tiroler Landesarchiv (TLA), Nachlässe Paul von Sternbach (NPvS), Box 1, Folder 2; Report by Heidegger regarding the trip to Switzerland with von Baron Sternbach, no date, and Report, April 1919, both in TLA, NPvS, Box 1, Folder 3.

78 Political Intelligence Department, Memorandum on Recent Events in Tyrol, Voralberg and Licthenstein, and their International Implications, 30 December 1918, TNA, FO 608-15.

79 Letter from the Bozen branch of the Tiroler Volkspartei to the provisional Assembly of Tyrol in Innsbruck, 3 January 1919, TLA, Referat Südtirol, Dokumentation Südtirolfrage, Box 24.

80 Minutes of the Obermännerkonferenz, March 24, 1919, TLA, NPvS, Box 1, Folder 3.

81 ‘Il sottocapo di stato maggiore dell’esercito, Badoglio, al Presidente del Consiglio, Orlando’, 10 May 1919’, document nr. 451, in Commissione per la Pubblicazione dei Documenti Diplomatici, *Documenti diplomatici italiani. Sesta serie: 1918–1922*, Vol. 3, 471.

82 Copy of cable n. 130 from the Comando Zona Trento to the Ministry of War, the Supreme Command of the Army and the General Civil Commissariat for Venezia Tridentina, 3 December 1919, in Archivio Centrale dello Stato (ACS), Ufficio Nuove Province (UNP), Box 58, Folder 10.

83 Mario Toscano, *Alto Adige – South Tyrol: Italy’s Frontier with the German World* (Baltimore, MD 1975), 43; Trafojer, ‘Die innenpolitische’, 70–109. The proposal is also mentioned in the Italian mission in Innsbruck to the Central Office for the New Provinces, document n. 1030, in Notiziario politico-economico, 6 April 1920, ACS, UNP, Box 61, Folder 6. See also Lindley to Curzon, Situation in Salzburg and Tyrol, 3 February 1921, C 2657/2657/3, TNA, FO, 371-5780.

84 Dennison Rusinow, *Italy’s Austrian Heritage, 1919–1946* (Oxford, 1969), 65–70.

85 ‘Die Friedenskonferenz vor dem Zusammenbruche’, *Der Tiroler*, 27 April 1919.

86 ‘Weihelose politische Nacht 1919’, *Der Tiroler*, 25 December 1919.

87 ‘Der frühere ital. Ministerpräsident Nitti über den Frieden’, *Der Tiroler*, 13 October 1920.

88 ‘Südtirol’, *Der Tiroler*, 24 October 1920.

89 For instance, secret schools. See Maria Villgrater, *Katakombenschule: Faschismus und Schule in Südtirol* (Bozen 1984), 532.

90 Steurer, *Südtirol*, 256–304; Steininger, *Südtirol*, 137–52.

91 Notably Storm and Ginderachter, ‘Questioning’, 753. See also Manela, *The Wilsonian*.

